# FMRP S499 Is Phosphorylated Independent of mTORC1-S6K1 Activity

**DOI:** 10.1371/journal.pone.0096956

**Published:** 2014-05-07

**Authors:** Christopher M. Bartley, Rachel A. O’Keefe, Angélique Bordey

**Affiliations:** 1 Departments of Neurosurgery, and Cellular and Molecular Physiology, Yale University School of Medicine, New Haven, Connecticut, United States of America; 2 Medical Scientist Training Program, Yale University School of Medicine, New Haven, Connecticut, United States of America; 3 Department of Neurobiology, Yale University School of Medicine, New Haven, Connecticut, United States of America; Université de Technologie de Compiègne, France

## Abstract

Hyperactive mammalian target of rapamycin (mTOR) is associated with cognitive deficits in several neurological disorders including tuberous sclerosis complex (TSC). The phosphorylation of the mRNA-binding protein FMRP reportedly depends on mTOR complex 1 (mTORC1) activity via p70 S6 kinase 1 (S6K1). Because this phosphorylation is thought to regulate the translation of messages important for synaptic plasticity, we explored whether FMRP phosphorylation of the S6K1-dependent residue (S499) is altered in TSC and states of dysregulated TSC-mTORC1 signaling. Surprisingly, we found that FMRP S499 phosphorylation was unchanged in heterozygous and conditional *Tsc1* knockout mice despite significantly elevated mTORC1-S6K1 activity. Neither up- nor down-regulation of the mTORC1-S6K1 axis *in vivo* or *in vitro* had any effect on phospho-FMRP S499 levels. In addition, FMRP S499 phosphorylation was unaltered in S6K1-knockout mice. Collectively, these data strongly suggest that FMRP S499 phosphorylation is independent of mTORC1-S6K1 activity and is not altered in TSC.

## Introduction

Altered mTOR signaling is a shared feature of many neurodevelopmental disorders that display high rates of mental retardation with comorbid autistic features such as TSC and fragile X syndrome (FXS) [1]. TSC, the canonical mTORopathy, is a monogenetic disorder due to mutations in *TSC1* or *TSC2*, which are upstream regulators of mTOR kinase activity in mTOR complex 1 (mTORC1). Most patients are born heterozygous for either *TSC* gene and experience additional inactivating mutations during development leading to loss of heterozygosity [2]. Subsequent runaway mTORC1 activity underlies cortical malformations and slow growing tumors. Although these malformations and associated seizure activity contribute to impaired cognition, imaging and neurocognitive studies suggest that they are not sufficient to fully explain the cognitive impairment in TSC patients [3], [4]. This notion is supported by animal models, in which *Tsc1* or *Tsc2* heterozygosity is sufficient to impair neuroplasticity and learning and memory despite the absence of brain malformations and clinical seizures [5]–[7]. Learning and memory impairments in juvenile *Tsc2* ^+/−^ mice are rescued by rapamycin treatment indicating that these deficits are reversible and mTORC1-dependent. In adult *Tsc2* ^+/−^ mice, an ERK inhibitor, but not rapamycin, rescued plasticity-dependent deficits, which is consistent with increased ERK activity in these mice [8]. Adult conditional *Tsc1* ^+/−^ mice, however, do not display increased ERK activity [9], suggesting cognitive deficits in these mice might be mTORC1-dependent. Collectively, these data suggest a biochemical contribution to cognitive deficits in TSC. One candidate molecule that is reportedly downstream of mTORC1 and involved in neuroplasticity is the fragile X mental retardation protein (FMRP), and could therefore contribute to TSC symptoms.

FMRP is an mRNA-binding protein that regulates the translation of ∼4–6% of brain mRNAs, many of which are involved in neuroplasticity [10]–[12]. Mutation of *FMR1* (the X-linked gene encoding FMRP) results in FXS, the leading cause of inherited intellectual disabilities and autism [13]. In the absence of FMRP, FXS model mice exhibit elevated mTORC1 activity, which may contribute to cognitive deficits and altered plasticity [14]. Under normal conditions, FMRP’s contribution to neuroplasticity is in part dictated by phosphorylation of serine 499 (S499), resulting in FMRP association with stalled polyribosomes and translational repression of synaptic mRNA [15], [16]. Interestingly, the kinase responsible for S499 phosphorylation was identified as the mTORC1-dependent kinase S6K1 [17]. S6K1 is thus a pivotal kinase linking mTORC1 activity to FMRP phosphorylation and function.

Because FMRP is absent in FXS and would be predicted to be hyperfunctional in TSC, it has been hypothesized that S6K1-dependent FMRP S499 hyperphosphorylation in TSC might explain some of the opposite phenotypes observed in these two models of autism [6], [18], [19]. We thus set out to investigate S6K1 activity as well as FMRP S499 phosphorylation in TSC mouse models. Surprisingly, we found that phospho-FMRP S499 (pFMRP) levels are unchanged in heterozygous and conditional *Tsc1* knockout mice despite significantly elevated mTORC1-S6K1 activity. Subsequent experiments revealed that neither up- nor down-regulating mTORC1-S6K1 signaling activity *in vivo* or *in vitro* has any effect on pFMRP levels, indicating that the mTORC1-S6K1 pathway plays no role in regulating S499 FMRP phosphorylation.

## Results

### FMRP and pFMRP Antibody Validation

**Figure 1:**
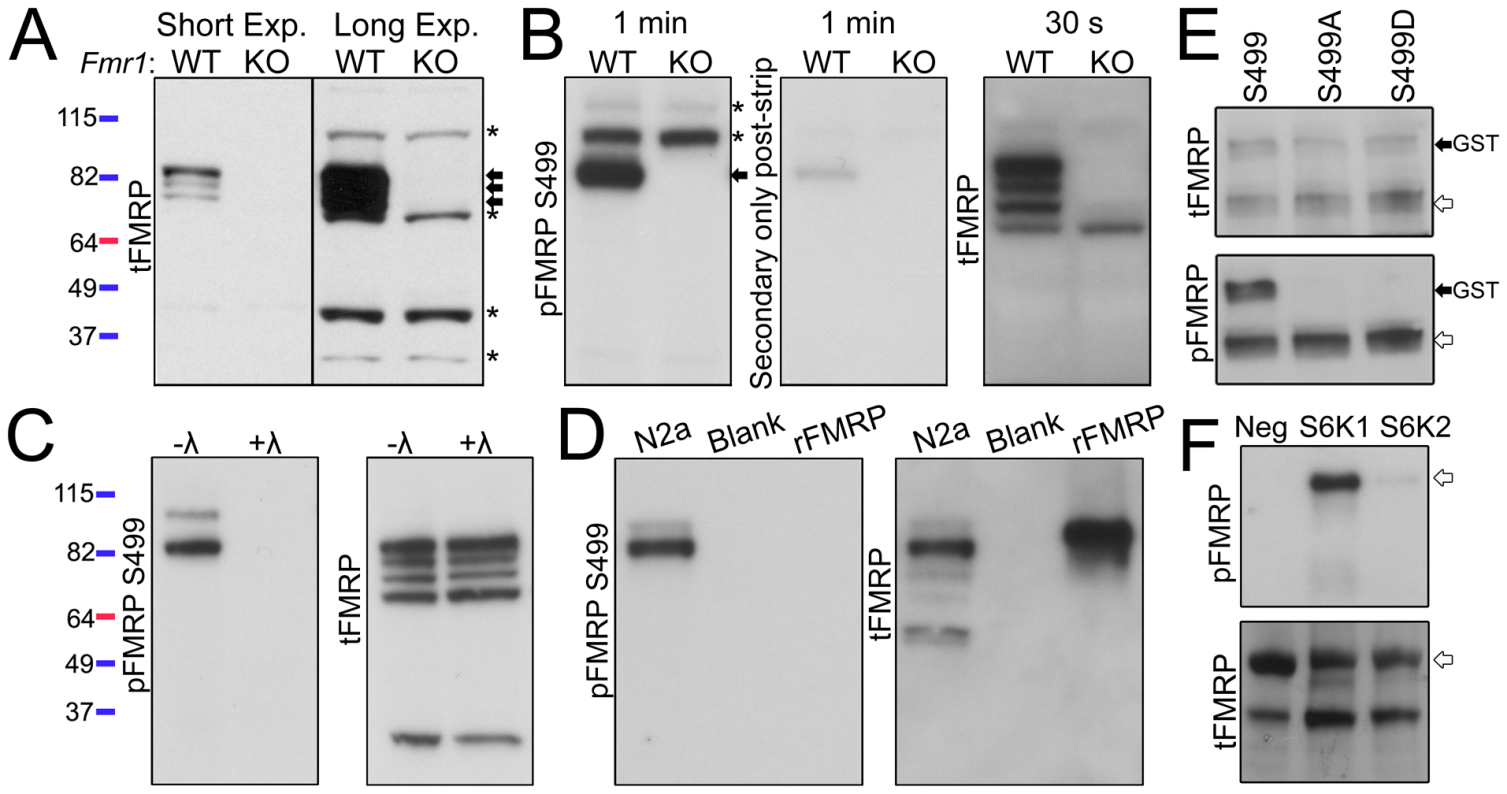
S6K1 phosphorylate FMRP S499 *in vitro*. (**A**) The specificity of the C-terminal, phospho-insensitive FMRP tFMRP antibody (Abcam #17722) was verified by immunoblotting whole cell cortical lysates from 2 month-old *Fmr1^y/+^* (WT) and *Fmr1^y/−^* (KO) mice. Short and long exposures (exp.) are shown. Arrows point to three FMRP-specific bands while asterisks point to nonspecific bands. Molecular weight is indicated by the colored ladders on the left which refer to panels A–D (**B**) *Fmr1^y/+^* and *Fmr1^y/−^* cortical lysates were immunoblotted with a pFMRP antibody and the film exposed for 1 minute (PhosphoSolution #p1125-499). The membrane was then stripped and reprobed with secondary only antibody (1 minute exposure) to verify removal of the primary antibody. The membrane was then probed with tFMRP antibody (30 second exposure). The arrow points to the FMRP-specific band while asterisks point to two nonspecific pFMRP bands. (**C**) Cortical lysate from *Fmr1^y/+^* mice was incubated with or without lambda (λ) phosphatase for 30 minutes and immunoblotted with pFMRP, stripped and reprobed with tFMRP. (**D**) Neuro2a cell lysate (N2a) and unphosphorylated recombinant human FMRP (rFMRP) were immunoblotted with pFMRP. (**E**) GST-FMRP S499, S499A and S499D were transfected into N2a cells and lysates analyzed by immunoblot 24 hours later. tFMRP recognized all three isoforms (black, GST-labeled arrow) as well as endogenous FMRP (white arrows) however pFMRP only recognized S499 and endogenous FMRP from the same membrane. (**F**) Recombinant FMRP was incubated with ATP and with, or without (Neg), S6K1 or S6K2 and immunoblotted for pFMRP or total FMRP (tFMRP = 1C3 antibody here). The experiments were reproduced in triplicate.

Prior to examining pFMRP levels, we validated the specificity of antibodies for total FMRP (tFMRP) and pFMRP S499 (referred to as pFMRP antibody). FMRP belongs to a small family of proteins that includes the fragile X-related proteins 1 and 2 (FXR1 and FXR2) and shares ∼70–80% homology with FXR1/2 in the N-terminal region but essentially no homology in the C-terminal region [20], [21]. Because some N-terminal antibodies can cross-react with FMRP-related proteins, we primarily utilized a C-terminal phospho-insensitive tFMRP antibody [15]. The tFMRP antibody recognized three distinct bands in cortical lysate from *Fmr1^y/+^* mice that were absent in *Fmr1^y/−^* mice (**Figure 1A**). Upon longer exposure nonspecific bands (marked with asterisks) were revealed indicating equal loading between lanes (**Figure 1A**). We tested two commercially available antibodies against pFMRP S499. One of the antibodies also recognized unphosphorylated FMRP and was not used further (data not shown). The second antibody (from PhosphoSolutions) has recently been used and validated [22]. We further characterized it as detailed below. The second antibody displayed a predominant pFMRP band that was absent in *Fmr1^y/−^* cortical lysate (arrow, **Figure 1B**). The pFMRP antibody also recognized two high molecular weight, non-specific bands (asterisks, **Figure 1B**). The same membrane was stripped (middle panel, **Figure 1B**) and reprobed with the tFMRP antibody indicating that the major pFMRP band was indeed FMRP (right panel, **Figure 1B**). To determine whether the pFMRP antibody is phospho-specific, *Fmr1^y/+^* cortical lysate was incubated with or without lambda phosphatase. The pFMRP antibody detected the FMRP-specific band in untreated lysate that was absent in phosphatase-treated lysate (left panel, **Figure 1C**). The same membrane was stripped and reprobed with the tFMRP antibody to ensure equivalent amounts of total FMRP in treated and untreated samples (right panel, **Figure 1C**). For additional verification the pFMRP antibody was tested against Neuro2a cell lysate (positive control, N2a) and recombinant human FMRP (rFMRP), which is devoid of post-translational modifications [23]. After running the gels for additional time to allow for better separation of FMRP isoforms, we found that the pFMRP antibody recognized two of four N2a FMRP bands, but did not recognize unmodified rFMRP (**Figure 1D**). Of the 12 predicted murine FMRP isoforms, only two (isoforms 1 and 7) contain the S499 phosphorylation site [24]. To validate that the pFMRP 499 antibody specifically recognized the S499 site, we obtained a vector encoding GST-tagged FMRP and generated two S499 mutants, one with an alanine (S499A) and one with an aspartic acid (S499D) substitution. In transfected N2a cells, the tFMRP antibody recognized all three GST-tagged FMRP proteins, but the pFMRP antibody only recognized the S499, and not S499A or S499D, GST-tagged FMRP proteins (**Figure 1E**). These data indicate that the pFMRP antibody specifically recognizes phosphorylated FMRP S499.

We next verified that S6K1 can phosphorylate FMRP S499 *in* *vitro* as previously reported [17]. Recombinant S6K1 or S6K2 were incubated with ATP and rFMRP. S6K1 displayed robust kinase activity towards FMRP as assessed by pFMRP immunoblotting (n = 3, **Figure 1F**). S6K2 exhibited minor activity towards FMRP compared to S6K1.

### Phospho-FMRP S499 is not Increased in *Tsc1* ^+/−^ Mice Despite Elevated S6K1 Activity

**Figure 2:**
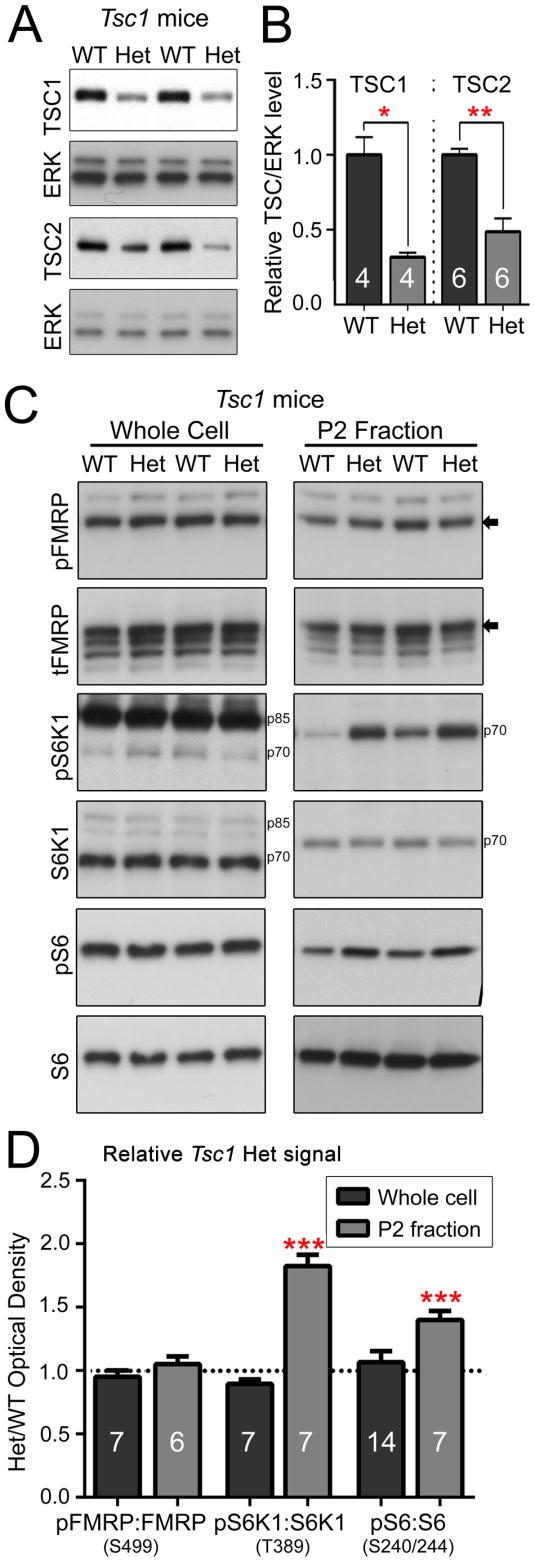
mTORC1 signaling, but not pFMRP S499 or tFMRP, is increased in *Tsc1* ^+/−^ mice. (**A**) Whole cell cortical lysates from 2 month-old male *Tsc1^+/+^* (WT) and *Tsc1^+/−^* (Het) mice were immunoblotted for TSC1 and TSC2. (**B**) TSC1:ERK and TSC2:ERK are significantly decreased in Het samples. The numbers in the bars indicate the number of mice per condition. (**C**) Whole cell and P2 fraction lysates from 2 month-old male *Tsc1^+/+^* and *Tsc1^+/−^* mice were analyzed by immunoblot. The arrows to the right of the blots indicate the pFMRP isoform. S6K1 isoforms are indicated by p70 and p85. (**D**) Quantification of western blot performed in whole cell lysate (dark grey) and P2 fraction (light grey) shown in (C). All bars represent Het phospho:total protein values normalized to WT control values. As such, WT values = 1 (as indicated by the dotted line). Error bars are SEM. Red asterisks indicate statistical significance (*P<0.05; ** *P*<0.01, and ***P<0.001) by one-sided (in B) and two-sided (in D) Mann-Whitney Tests, N are listed on the bar graphs.

Because mTORC1 pathway activation has not been thoroughly characterized in *Tsc1* ^+/−^ mice, we first examined the phosphorylation levels of the mTORC1-dependent site on S6K1 T389 (pS6K1), which functions as an activating phosphorylation. We secondarily characterized phosphorylation of the S6K1-dependent sites on ribosomal protein S6 S240/244. We used the hippocampus of 2 month-old male *Tsc1* ^+/−^ mice to avoid potential variations in tFMRP levels between gender [25], [26]. For all the conditions, we compared and graphed the levels of phospho-protein divided by total protein (*e.g*., pFMRP:tFMRP). Despite significantly decreased levels of TSC1 and TSC2 in *Tsc1* ^+/−^ compared to *Tsc1* ^+/+^ mice (N = 4 for TSC1 and 6 for TSC2, **Figure 2A and B**), pS6K1 and pS6 levels were identical between *Tsc1* ^+/+^ and *Tsc1* ^+/−^ mice in whole cell lysates (N = 6–14, **Figure 2C and D**, dark grey bars in D). Significance (asterisks) is noted on each graph and the statistical tests are mentioned in each figure legend. We thus prepared synaptically enriched P2 fractions [27] to more specifically examine neuronal pS6K1 and pS6 levels. In P2 fractions, pS6K1 as well as pS6 were significantly elevated in *Tsc1* ^+/−^ mice compared to *Tsc1* ^+/+^ mice, suggesting that mTORC1-S6K1 activity is significantly elevated in neurons of *Tsc1* ^+/−^ mice (N = 6 or 7, **Figure 2C and D**, light grey bars in D). Surprisingly, however, despite elevated phosphorylation of mTORC1-dependent signaling markers, pFMRP levels were not significantly different between the P2 fractions of *Tsc1* ^+/+^ and *Tsc1* ^+/−^ mice (**Figure 2C and D**). These data led us to question and further examine the mTORC1-S6K1 connection to FMRP phosphorylation.

### Phospho-FMRP S499 is Not Altered in the Conditional *Tsc1* ^−/−^ Forebrain or Downstream of Hyperactive mTORC1 *in vitro*

**Figure 3:**
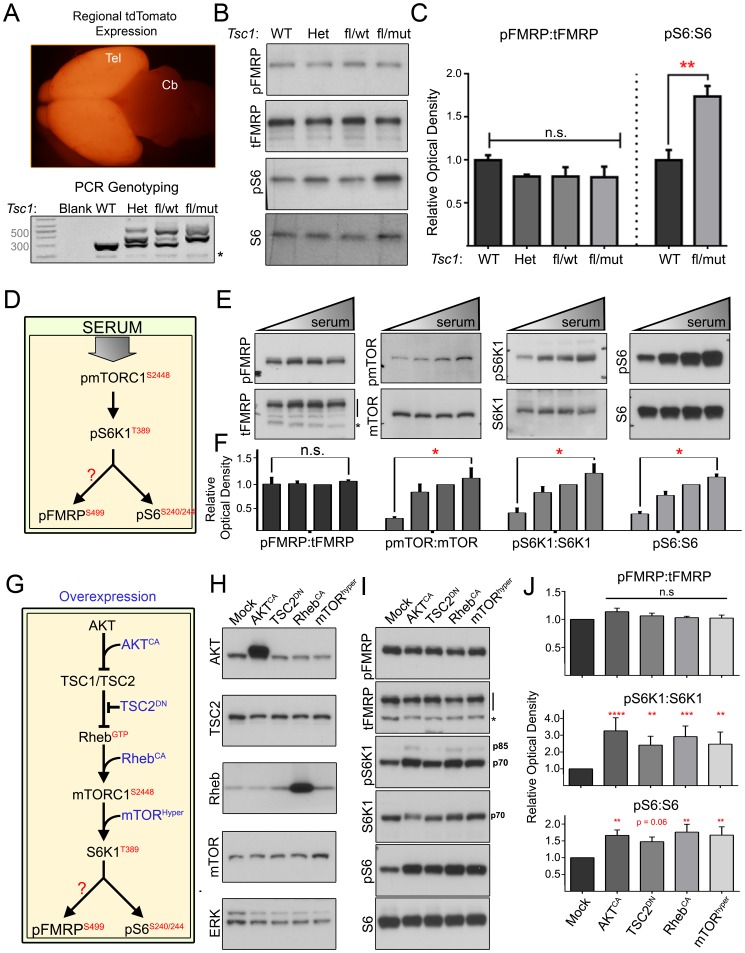
Hyperactive mTORC1-S6K1 does not alter pFMRP S499. (**A**) Cre expression and genetic recombination were verified by region-specific tdTomato expression (note tdTomato in telencephalon, Tel, but not cerebellum, Cb). Genotypes were confirmed by PCR: wild type mice (WT) have two wild type alleles (wt, 295bp amplicon), heterozygous mice (Het) have one wt and one mutant allele (mut, 370bp amplicon), and conditional mice have one floxed allele (fl, 480bp amplicon) with either a wt or mut allele. Asterisks indicate nonspecific bands (**B**) Immunoblotting of cortical lysates from P7 *Tsc1* mice of different genotypes listed under the blots. (**C**) There is no statistical difference in pFMRP:tFMRP among genotypes despite significantly increased pS6:S6 in *Tsc1* ^fl/mut^ vs. *Tsc1* ^WT^. pFMRP:tFMRP was quantified by unpaired, one-way ANOVA. pS6:S6 was quantified using unpaired, nonparametric one-sided Mann-Whitney test (** = p<0.01). Error bars STDEV. N = 3. (**D**) Model of serum-mediated mTORC1 pathway activation. (**E**) Immunoblots for pFMRP, pmTOR, pS6K1 and pS6 suggest that although the mTORC1 pathway responds to serum, pFMRP is not altered. N2a were cells maintained in 5% serum were transferred to 0, 2.5, 5 or 10% serum 1 hour prior to lysis. (**F**) Bar graphs of the phospho:total protein ratio shown in (E) showing that pFMRP:tFMRP is unchanged despite significantly increased mTORC1 pathway activity. Unpaired, nonparametric one-sided Mann-Whitney tests compared 0 and 10% serum conditions for each phospho-protein. We used a one-sided test because increased mTORC1 pathway activity is expected following serum application. Error bars = SEM. N = 4 per condition. (**G**) Model of mTORC1 pathway activation by overexpression. (**H**) Immunoblotting verified overexpression of transfected genes with the exception of TSC2^DN^ whose large C- and N-terminal deletions render it unrecognizable to many antibodies. N2a cell lysates were collected 48 hours post-transfection. (**I**) Immunoblotting for pFMRP, pS6K1, pS6 and their total protein counterparts. (**J**) Quantification of phospho:total protein ratios normalized to mock transfected cells – unpaired, one-way ANOVA corrected for multiple comparisons. P values were derived from post-hoc Dunnett’s test (*, **, *** & **** = P≤0.05, 0.01, 0.001 and 0.0001 respectively). N = 4 per condition.

To determine whether FMRP phosphorylation might be increased in the *Tsc1* ^−/−^ state *in vivo*, we generated conditional heterozygous and knockout *Tsc1* mice. *Tsc1^fl/+^;R26R-*tdTomato (fl for floxed) mice were bred against *Tsc1^wt/−^;Emx1-*Cre mice to yield mixed litters of forebrain-specific *Tsc1* wildtype, heterozygous, and knockout mice. In *Emx1*-Cre mice, Cre recombinase is expressed in forebrain glial and glutamatergic progenitors beginning around E9.5 [28]. Forebrain-specific Cre-mediated recombination was verified by the expression of tdTomato fluorescence and PCR for *Tsc1* (**Figure 3A**). mTORC1-S6K1 signaling was dramatically elevated in the forebrain as demonstrated by significantly increased S6 S240/244 phosphorylation in *Tsc1* ^fl/−^;*Emx1*-Cre mice containing *Tsc1* ^−/−^ cells. Despite significantly elevated mTORC1-S6K1 activity, neither tFMRP nor pFMRP levels were altered in the cortex of these mice (**Figure 3B and C**).

Because mTORC1 could regulate FMRP S499 phosphorylation independent of TSC1/2, we next used several manipulations to increase mTORC1 activity in N2a cells and assessed the impact on pFMRP levels. Serum is known to activate the mTORC1 pathway (**Figure 3D**). N2a cells were thus exposed to 0, 2.5, 5 or 10% serum for 1 hour prior to sample collection. Using mTOR S2448 [29], S6K1 T389 and pS6 S240/244 as readouts of mTORC1 signaling, we found that mTORC1 pathway activity was highly correlated with serum concentration in N2a cells. In contrast, pFMRP levels were unaffected by serum (n = 4 per condition, **Figure 3E and F**). We then overexpressed different components of the mTORC1 pathway that are expected to increase mTORC1 activity: constitutively active AKT (AKT^CA^), dominant negative TSC2 (TSC2^DN^), constitutively active Rheb (Rheb^CA^), and hyperactive mTOR (mTOR^hyper^) (**Figure 3G and H**). All of these manipulations led to a significant increase in S6K1 T389 and pS6 S240/244 phosphorylation, however, pFMRP levels remained unchanged in these same samples (n = 4 per condition, **Figure 3I and J**).

### Pharmacological Inhibition of mTORC1 and S6K1 Activity does not Affect pFMRP S499 Levels *in vitro*

**Figure 4:**
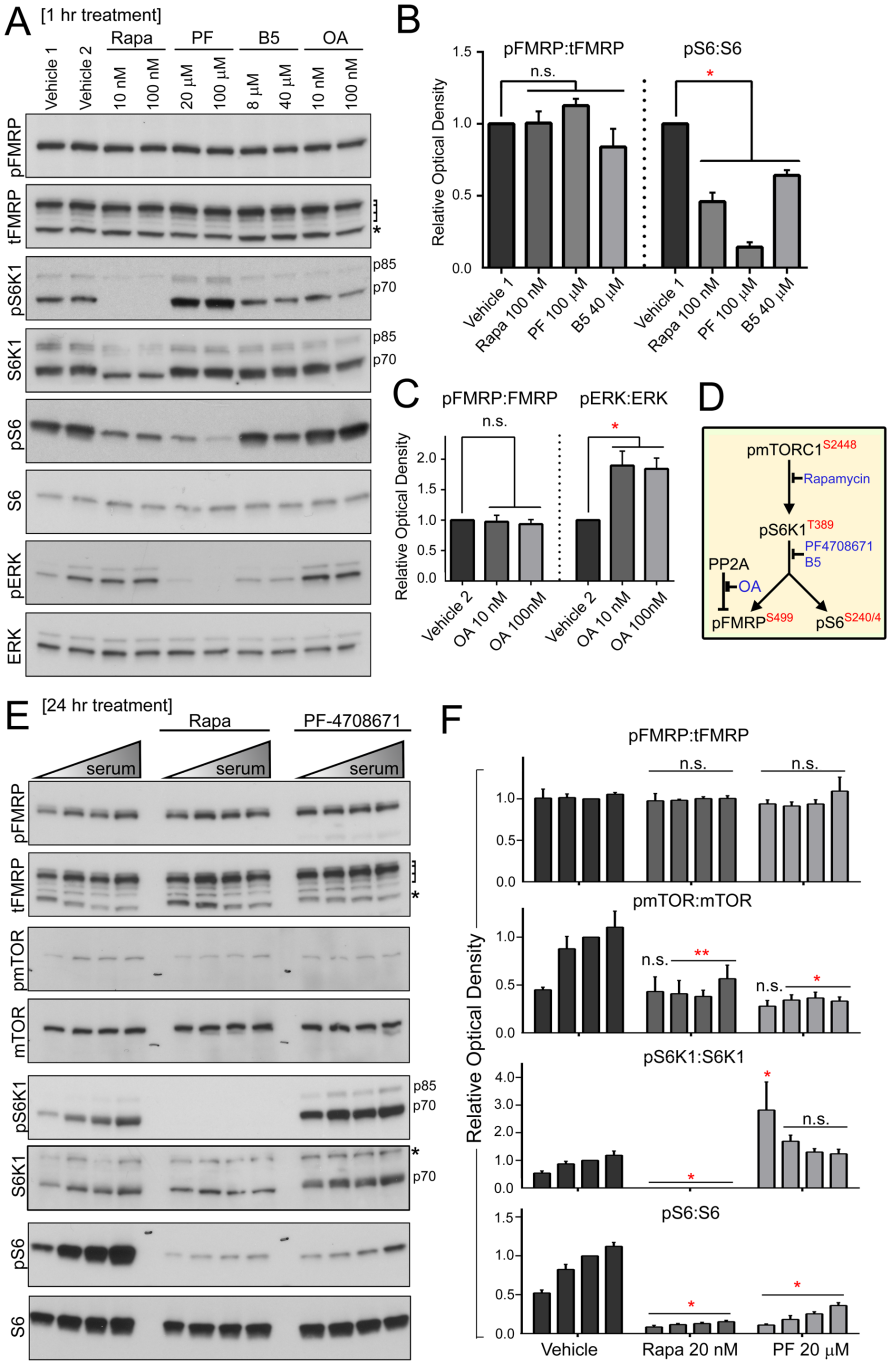
Inhibiting mTORC1 and S6K1 activity has no effect on pFMRP S499 levels *in vitro*. (**A**) N2a cells were treated with vehicle 1 (DMSO), vehicle 2 (ethanol), rapamycin (Rapa), PF-4708671 (PF), bisindolylmaleimide V (B5) or okadaic acid (OA) for 1 hour prior to cell lysis followed by immunoblotting for pFMRP, pS6K1, pS6, pERK T202/Y204 (readout for OA), and their total protein counterparts. Laddered bracket to right of tFMRP indicates FMRP isoforms and the asterisk denotes a nonspecific band. S6K1 isoforms are marked by p80 and p75. (**B and C**) Statistical verification of stable pFMRP:tFMRP across all conditions despite a significant decrease in pS6:S6 subsequent to mTORC1-S6K1 inhibition (B) and increase in pERK:ERK subsequent to PP2a inhibition (C). One way ANOVA with post-hoc Dunnett’s test (N = 4 per condition. Error bars = SEM). (**D**) Model of pathway and effect of pharmacological inhibitor. (**E**) N2a cells. Immunoblotting for pFMRP, tFMRP and mTORC1 pathway components from N2a cells maintained in 5% serum, transferred to increasing concentrations of serum (0, 2.5, 5 or 10%), and treated with vehicle (DMSO), rapamycin (Rapa) or PF-4708671 (PF) for 24 hours. Laddered bracket to the right of tFMRP blot indicates FMRP isoforms and the asterisk a nonspecific band. S6K1 isoforms are indicated by p85 and p70. The asterisk to the right of the total S6K1 blot indicates residual tFMRP signal from the blot above. (**F**) Bar graphs of (E). Statistical analysis: unmatched two-way ANOVA corrected for multiple comparisons with a post-hoc Tukey’s test. N = 4 per condition. Error bars = SEM.

To determine whether apparent insensitivity of pFMRP levels to elevated mTORC1 signaling was simply an artifact due to a ceiling effect, we inhibited mTORC1-S6K1 signaling *in vitro* and *in vivo.* We also inhibited PP2a *in vitro*, which was previously shown to dephosphorylate FMRP [30]. In total, three drugs were utilized to inhibit mTORC1-S6K1 signaling: rapamycin, PF-4708671, and bisindolylmaleimide V (B5). Rapamycin inhibits the mTORC1 complex and has been extensively characterized. PF-4708671 and B5 are recently identified S6K1 inhibitors [31], [32]. Okadaic acid was used to inhibit PP2a activity (**Figure 4D** for signaling model). We consistently found that rapamycin, PF-4708671, and B5 significantly reduced the activity of mTORC1-S6K1 signaling as assessed with pS6 S240/244 even in conditions of serum stimulation (**Figure 4A, B, E and F**). As previously reported, PF-4708671 induced a paradoxical hyperphosphorylation of S6K1 T389 [31] (**Figure 4A and E**). Okadaic acid significantly increased the phosphorylation of the PP2a substrate ERK T202/Y204 (**Figure 4A and C**). However, none of these blockers affected pFMRP or tFMRP levels (**Figure 4A–C and E-F**).

### Phospho-FMRP S499 is Unaltered by Pharmacological Inhibition of mTORC1 or S6K1 Activity *in vivo,* or in *S6k1*-knockout Mice

**Figure 5:**
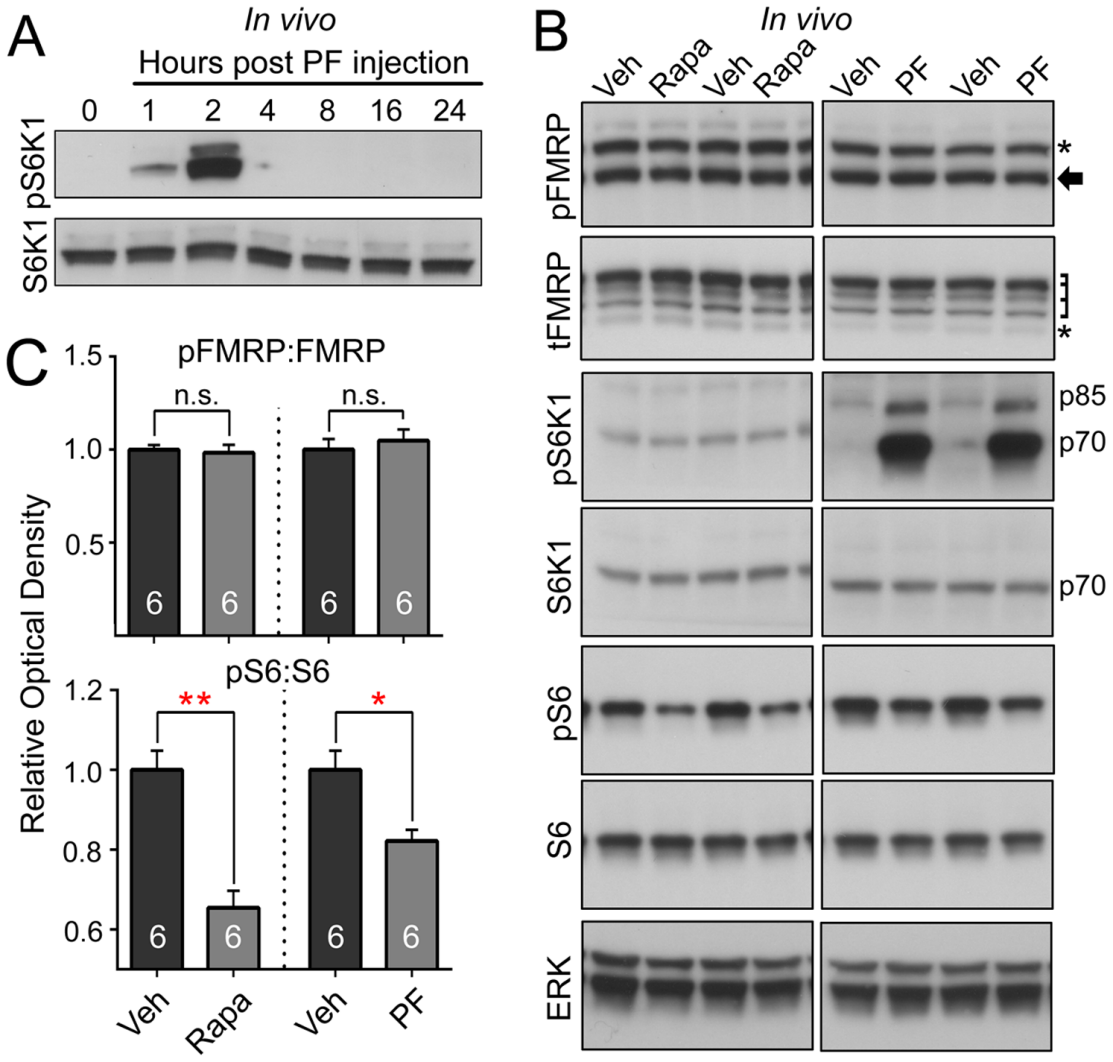
Inhibiting S6K1 does not alter pFMRP S499 levels *in vivo*. (**A**) CD1 mice were intraperitoneally injected (IP) with PF (75 mg/kg) and sacrificed at various time points thereafter. Hyperphosphorylation of S6K1 T389 (pS6K1) detected at 2 hours suggests that this compound can cross the blood brain barrier. N = 3 per time point. (**B**) Immunoblots from cortical lysates from CD1 mice treated IP with rapamycin (Rapa, 1.5 mg/kg for 5 days), PF-4708671 (PF, 75 mg/kg for 2 hours), and vehicle (DMSO) alone. Asterisks indicate nonspecific bands, the arrow indicates the pFMRP isoform, laddered bracket indicates the tFMRP isoforms, and S6K1 isoforms are indicated by p85 and p70. (**C**) Quantification verifies a significant decrease in pS6:S6 but no change in pFMRP:FMRP following mTORC1 or S6K1 inhibition *in vivo*. *P<0.05 and ** *P*<0.01 by unpaired, one-sided Mann-Whitney Test. A one-sided test was used considering that decreased pS6 levels were expected. N = 6 per condition. Error bars = SEM.

For *in vivo* experiments, we initially determined that PF-4708671 crosses the blood-brain-barrier, and found that it was cleared from the brain by 4 hours post-injection (**Figure 5A**). We performed intraperitoneal injections of either rapamycin (1.5 mg/kg) or PF-4708671 (75 mg/kg) while control mice were injected with a similar volume of vehicle (*i.e*, DMSO). Rapamycin injections were performed daily for 5 days and PF-4708671 mice were injected once and sacrificed two hours post-injection. Both rapamycin and PF-4708671 significantly decreased mTORC1-S6K1 activity as demonstrated by reduced pS6 S240/244 levels, however, pFMRP levels were unchanged (**Figure 5B and C**).

**Figure 6:**
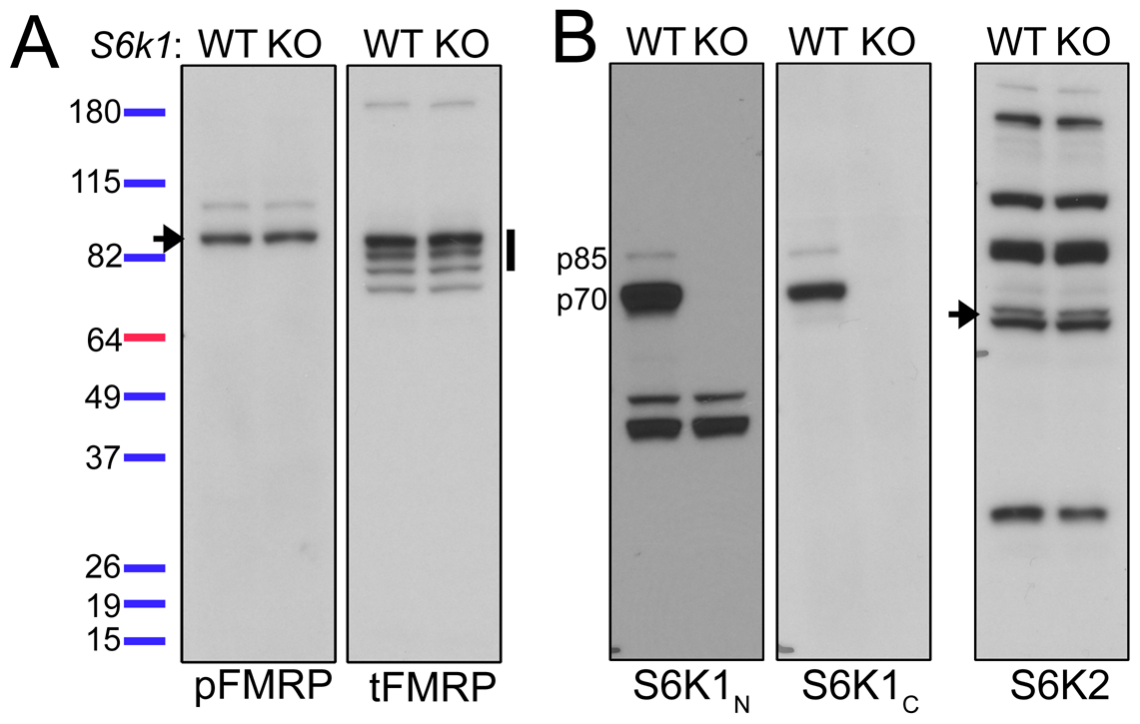
S6K1 activity is dispensable for phosphorylation of FMRP S499 *in vivo*. (**A and B**) Cortical lysates from male S6K1^WT^ (wild type) and S6K1^KO^ (knockout) mice were immunoblotted for pFMRP (arrow), tFMRP (vertical band) in (A), and N- and C-terminal S6K1 (S6K1_N_ and S6K1_C_, respectively) and S6K2 (arrow) in (B). N = 3 sets of mice.

It was previously reported that pFMRP S499 was absent in *S6k1* ^KO^ mice [17]. We thus probed *S6k1* ^+/+^ and *S6k1* ^−/−^ cortical lysates with N- and C-terminal S6K1 antibodies to verify the absence of *S6k1* full length and/or partial protein products. Despite a complete absence of S6K1 protein, neither pFMRP nor tFMRP levels were different between *S6k1* genotypes, suggesting that S6K1 is dispensable for phosphorylation of native FMRP at S499 (**Figure 6A and B**).

Collectively, these data strongly suggest that FMRP S499 is not phosphorylated by S6K1 and demonstrate that S6K1 is not required for FMRP S499 phosphorylation.

### mTORC1 Activity is not Required for mGluR I-dependent Phosphorylation of FMRP S499

**Figure 7:**
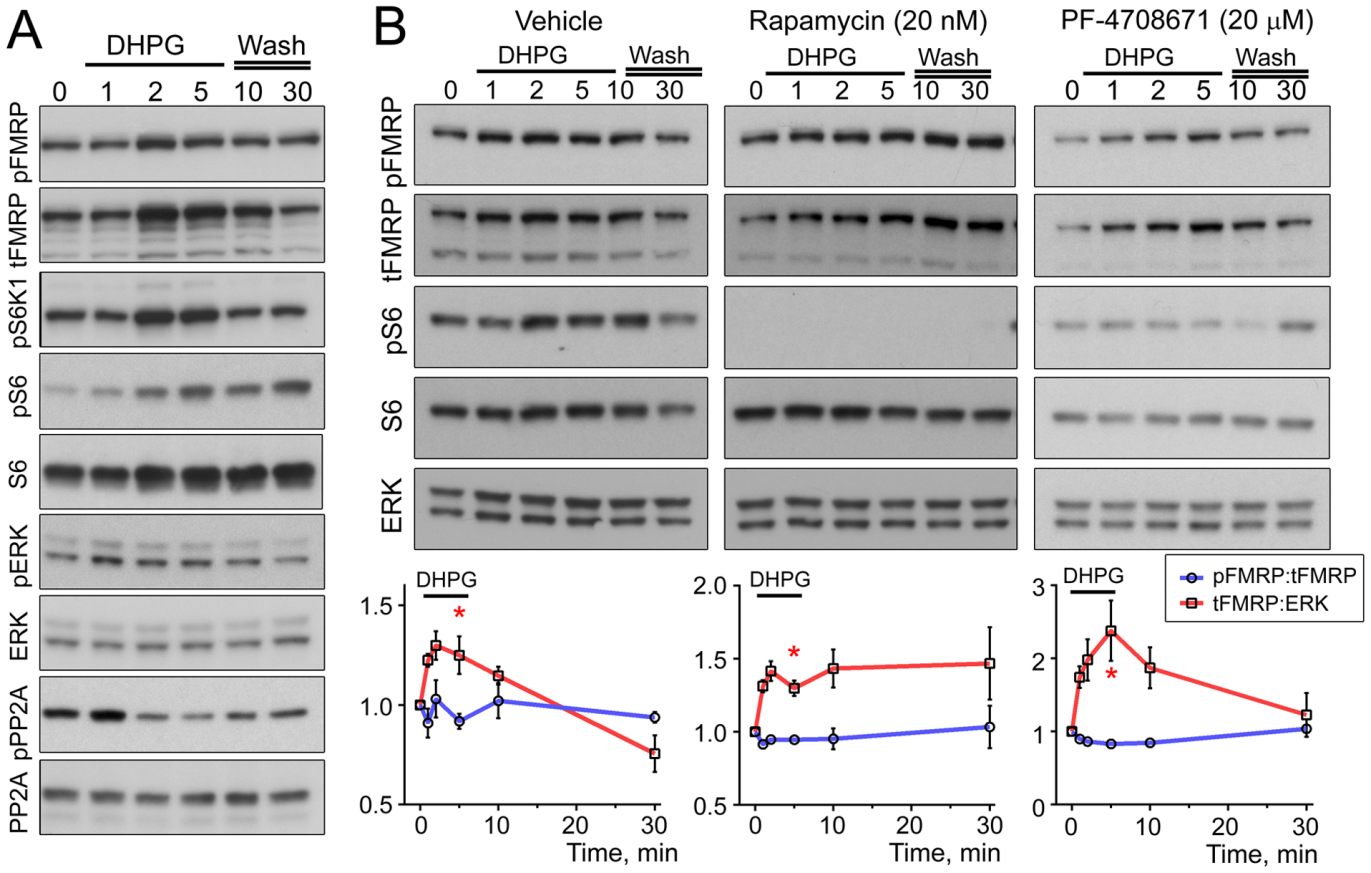
mGluR I stimulation does not increase FMRP S499 phosphorylation. (**A**) Immunoblots from N2a cells treated with (S)-DHPG (100 µM) for 1, 2 or 5 minutes. The lysates were collected after 1, 2 or 5 minutes of DHPG treatment and after 5 or 25 minutes washout following the 5 minutes DHPG treatment. Cells were maintained in 10% serum. The media was replaced with 5% serum 2 hours prior to DHPG application (n = 6). (**B**) Immunoblots using similar protocol as in (A) but with 1 hour drug pretreatment. Drugs were vehicle (DMSO), rapamycin (Rapa, 20 nM) or PF-4708671 (PF, 20 µM). (**C**) Quantification of (B) for pFMRP:tFMRP and tFMRP:ERK normalized to their respective baseline at time 0. One-sided Mann-Whitney tests comparing data at 5 min to time 0 was used since tFMRP was expected to increase, * = P<0.05, n = 3 per condition. Error bars = SEM.

Given that mGluR class I activity has been reported to regulate the phosphorylation of FMRP S499 [17], we investigated the dynamics of FMRP translation and phosphorylation following mGluR I activation in N2a cells which, have been used by others to study mGluR I-dependent FMRP signaling [33]. N2a cells were treated with (S)-DHPG (100 µM) for 0, 1, 2, and 5 minutes. Additional samples were collected 5 and 25 minutes post-washout. Importantly, DHPG elicited dynamic changes in pERK1/2 and mTOR-S6K1 signaling (**Figure 7A**). tFMRP levels increased as early as 2 minutes following DHPG application and was accompanied by increased FMRP S499 phosphorylation, but there was no change in the ratio of pFMRP:tFMRP (n = 6, **Figure 7A**). In addition, experiments in the presence of rapamycin or PF-4708671 did not prevent FMRP phosphorylation accompanying FMRP synthesis (n = 3 each, **Figure 7B**).

## Discussion

Our study demonstrates that mTORC1-S6K1 signaling does not contribute to phosphorylation of FMRP S499.

In order to study the mTORC1-S6K1-FMRP connection, we first examined whether mTORC1 pathway activity is increased in *Tsc1* ^+/−^ mice. Of note, our data represent the first biochemical detection of elevated S6K1 T389 phosphorylation in *Tsc1* ^+/−^ mice. In these mice, we only detected elevated S6K1 T389 in neuronally enriched P2 fractions supplemented with okadaic acid. Given that mTORC1-S6K1 signaling was found not to be elevated in the *Tsc1^−/−^* astrocytes [34], the lack of elevated S6K1 phosphorylation in whole hippocampal lysate could be due to a masking effect of glial mTORC1 signaling, which is presumably normal in the heterozygous state. Consistent with elevated S6K1 T389 phosphorylation, we also observed an increase in phosphorylation of the S6K1 substrate S6 S240/244 in P2 fractions. While one group reported elevated S6 S235/236 in *Tsc2* ^+/−^ mice [5], phosphorylation of S235/236 can be modulated independent of mTORC1 signaling [35]. Additionally, although pS6 S235/236 was elevated in the telencephalon of *Tsc2*ΔRG mice (dominant negative), this was found to be due to enhanced ERK-RSK signaling rather than mTORC1 activity [9]; ERK signaling, however, is not elevated in conditional *Tsc1* ^+/−^ telencephalon [9]. Although elevated pS6 S240/244 phosphorylation could have been due to decreased phosphatase activity, we found that expression of the main pS6 phosphatase, PP2a, was elevated in our P2 fractions making this explanation less likely (unpublished observations, Bartley and Bordey).

Importantly, despite evidence of elevated mTORC1-S6K1 activity in P2 fractions of *Tsc1^+/−^* mice and in the forebrain of conditional *Tsc1* knockout mice, we could not detect elevated FMRP S499 phosphorylation. Because we found that S6K1 can phosphorylate recombinant human FMRP *in vitro,* we tested whether our inability to detect increased FMRP S499 phosphorylation may be because the mTORC1-S6K1 axis modulates FMRP S499 phosphorylation independent of the TSC pathway or because basal FMRP S499 phosphorylation is saturated. We therefore tested whether either overexpression of other inputs to mTORC1 or inhibition of mTORC1-S6K1 activity would alter FMRP S499 phosphorylation.

None of the manipulations that altered mTORC1-S6K1 activity, either *in vitro* or *in vivo*, affected the degree of FMRP S499 phosphorylation. Finally, FMRP S499 phosphorylation is identical between *S6k1* ^+/+^ and *S6k1* ^−/−^ mice, indicating that S6K1 is dispensable for normal FMRP S499 phosphorylation. This finding is in contrast with data in a previous study [17]. Although our study used a different phospho-specific antibody, the antibody used by the other group was validated against FMRP S499A in two other studies [30], [36]. It is possible that their antibody recognizes other, non-S499, phosphorylated motifs on FMRP. If a negative charge at S499 is required for phosphorylation of other sites on FMRP, as suggested by radiolabeling studies using S499, S499A and S499D [16], then the non-specificity of their antibody would not be identified using FMRP S499A (which would be predicted to be completely unphosphorylated). In any event, consistent with findings from other groups, we found that mGluR I stimulation by DHPG does increase the level of tFMRP [37]–[41]. We also found that pFMRP S499 increased in parallel with tFMRP following DHPG stimulation, but was insensitive of mTORC1-S6K1 inhibition.

Collectively, these data demonstrate that S6K1 is not required for phosphorylation of endogenous FMRP at S499. Furthermore, these data indicate that the mTORC1-S6K1 pathway does not regulate the phosphorylation of FMRP S499 in any way. This negative finding is consistent with the fact that the sequence surrounding FMRP S499, EASNApS, contains none of the features that would render it a good S6K1 candidate. The preferred phosphorylation motif for S6 kinases is relatively well preserved, RXRXXpS (where X = any amino acid and p denotes the phosphorylated residue). With rare exception S6 kinase substrates deviate from this sequence [42], [43], however, sequences that do diverge generally contain an arginine (R) in the −3 or −5 position [44]. Although we and another group found that S6K1 can phosphorylate FMRP *in vitro* [17], this may be an artifact of the high concentrations of isolated S6K1 and FMRP utilized in these kinase assays which could promote a nonphysiologic interaction between these two proteins. In the previous study, a phospho-specific antibody was used to verify that S6K1 can phosphorylate FMRP. However it should be noted that in this study activity- and S6K1-dependent changes in phosphorylated FMRP were only measured using radioactive phosphate. This radioactive method, however, cannot distinguish phosphorylated S499 from other phosphorylated sites, which could be S6K1-dependent. We cannot explain, however, why pFMRP S499 was absent from S6K1 knockout mice using their phospho-specific antibody but present in our S6K1 knockout mice.

Our findings open clear questions. In particular, it is important to identify the kinase(s) responsible for FMRP S499 phosphorylation. The recent finding that phospho-mimetic FMRP but not phospho-dead FMRP can fully rescue *dFmr1* drosophila highlights the biological significance of this phosphorylation site [45]. A previous study in drosophila reported that CK2, formerly casein kinase 2, phosphorylates a S499 homologous site, dFMRP S406 [46]; and this has been suggested in mice by [47] as well as in our hands (Bartley and Bordey, unpublished observations). If CK2 is the kinase for mammalian FMRP S499 this poses a particular conundrum for the activity-dependent regulation of FMRP S499 phosphorylation. CK2 is considered to be a constitutively active kinase which would suggest that S499 is regulated primarily by phosphatase activity or that phosphorylation sites other than S499 are regulated in an activity-dependent manner. In general, activity-dependent detection of changes in the phosphorylation of FMRP has been performed using radioactive phosphate or phospho-serine antibodies, which are incapable of distinguishing S499 phosphorylation from other phosphorylated residues.

Our initial interest was to investigate the contribution of dysregulated FMRP S499 phosphorylation to TSC. To our surprise we were unable to find a link between mTORC1-S6K1 and S499 phosphorylation under any condition. Although our findings strongly suggest that another kinase is responsible for the phosphorylation of FMRP S499, the mTORC1-S6K1 pathway may yet regulate the phosphorylation of other FMRP residues.

## Materials and Methods

### Ethics Statement

All animal research protocols were approved by the Institutional Animal Care and use Committee, Yale University.

### Animals

We used male wild-type and transgenic mice except for conditional *Tsc1* ^−/−^ mice (see description below). *Tsc1^+/−^* mice (+ for wildtype (wt) and - for mutant (mut) alleles, NCI), also noted *Tsc1* ^Het^ in figure legends, were generated by David J. Kwiatkowski (Brigham and Women’s Hospital, Harvard Medical School, Cambridge, Massachusetts, USA) and were of mixed background: B6;129S4, C57BL/6J, BALB/cJ and 129SvJae. *Fmr1^y/−^* and *Fmr1^y/+^* mice, which are knockout (KO) and WT mice, respectively, were a gift from Dr. Leonard Kaczmarek, Yale University, New Haven, CT. *S6k1^+/+^* and *S6k1^−/−^* samples (also WT and KO) were a gift from Dr. Kat Takeda, National Jewish Health, Denver, CO. To generate forebrain-specific conditional *Tsc1^−/−^* (*Tsc1* ^KO^) mice, we bred *Tsc1^flox/+;^Emx1-Cre^−/−^; R26R-*tdTomato*^+/+^* (where +/+ connotes the presence of the tdTomato gene) to *Tsc1^+/−^;Emx1-Cre^+/+^;R26R-*tdTomato*^−/−^ mice*. *Emx1*-Cre mice were kindly provided by Dr. Cardin (Department of Neurobiology, Yale University, originally from Jackson labs). *R26R-*tdTomato mice were obtained from the Jackson Labs. *Tsc1^fl/+^* mice (Jackson Labs) were also generated by David J. Kwiatkowski. With the exception of *Tsc1:Emx1-*Cre transgenic mice, all mice used in this study were 2 months old. Because all *Tsc1^flox/−^* mice die by P20 due to seizure, only P7 mice were used in this study.

Genotyping was performed either *in house* or using Genetyper services (www.genetyper.com). For *in-house* and Genetyper-assisted genotyping we used a three-primer protocol that allows for the simultaneous detection of wild type, mutant and floxed *Tsc1* alleles: TSC1F4536∶5′ – AGG AGG CCT CTT CTG CTA CC; TSC1R4830∶5′ – CAG CTC CGA TGA AGT G; TSC1R6548∶5′ – TGG GTC CTG ACC TAT CTC CTA.

### Tissue Preparation

Mice were anesthetized with isoflurane followed by decapitation. Brains were acutely dissected in ice-cold Hank’s Balanced Salt Solution (HBSS, GIBCO Cat. No. 14170–112), snap frozen in liquid nitrogen and stored at −80°C. For whole cell lysates, samples were homogenized in RIPA buffer supplemented with DNase I (8 U/10 ml), 1x HALT protease/phosphatase inhibitor cocktail (Pierce #78443), and okadaic acid (100 nM). Protein concentrations were quantified using a standard BCA protein assay (Pierce #23225). For a given experiment, samples were diluted to the same concentration with lysis buffer and then boiled in an equal volume of 2x Laemmli buffer at 99°C for 5 minutes.

### P2 Fractionation

Tissues were homogenized in P2 lysis buffer (4 mM HEPES, 0.32 M Sucrose, 1x HALT, 5 mM EDTA, and 100nM Okadaic Acid) with micro-pestles (RPI #199222); 20 strokes/sample. Homogenates were centrifuged at 1000 g for 10 minutes at 4°C and the pellet discarded. Supernatants were recentrifuged at 10,000 g x 15 minutes at 4°C and the supernatant set aside as the cytoplasmic fraction. The pellet (P2 fraction) was resuspended in P2 buffer and centrifuged again at 10,000 g for 15 minutes at 4°C. The supernatant was discarded and the P2 fraction was resuspended in 50 mM Tris-H_2_O supplemented with HALT. Cytoplasmic and P2 fraction protein concentrations were quantified and the samples were boiled in an equal volume of 2x Laemelli Sample Buffer. We validated that P2 fractions were enriched for synaptic proteins PSD95 and SAPAP3 and relatively depleted of nonsynaptic proteins such as α–tubulin and the glial protein GFAP [48] (data not shown).

### Lambda Phosphatase Assay

A single CD1 adult mouse hippocampus was homogenized in 300 µl phosphatase assay lysis buffer (4 mM HEPES, 0.5% Triton-X-100, 120 mM NaCl, and 2 Roche protease inhibitor tablets per 10 ml). The sample was centrifuged at 13,000 rcf for 10 minutes at 4°C. 800U lambda phosphatase (NEB #P0753S) was added to 100 µl supernatant and the sample incubated at 37°C for 30 minutes. The reaction was terminated by addition of an equal volume of 2x Laemmli buffer.

### Antibodies

Antibodies and usage parameters are listed in Table 1.

**Table 1:** List of antibodies.

ANTIBODY	MANUFACTURER(Species) #Cat. No.	Primary;Secondary	Blocking
AKT	Cell Signal (Rb) #4685	1∶5,000; 1∶5000	5% Milk/TBST
pERK T202/Y204	Cell Signal (Rb) #4370	1∶10,000; 1∶5000	5%BSA/TBST
ERK	Santa Cruz (Rb) #sc-94	1∶20,000; 1∶10,000	5% Milk/TBST
pFMRP S499	PhosphoSolutions (Rb)#p1125-499	1∶1,000;1∶2,000	Block milk 5%, probe in BSA (pFMRPmust be probed for prior to FMRP)
FMRP	Abcam (Rb) #17722	1∶5,000;1:,5,000	5% Milk/TBST
FMRP 1C3	Millipore (Ms) #MAB2160	1∶2,000;1∶2,000	5% Milk/TBST
pmTOR S2448	Cell Signal (Rb) #5536	1∶50,000;1∶50,000	5%BSA/TBST
mTOR	Cell Signal (Rb) #2983	1∶1,25,000;1∶50,000	5% Milk/TBST
Rheb	Thermo (Rb) #PA5-20129	1∶3,000;1,2,000	5% Milk/TBST
pS6 S240/244	Cell Signal (Rb) #5364	1∶10,000;20,000	5% BSA/TBST (total S6 must be probedfor first as pS6 is not efficiently stripped)
Total S6	Cell Signal (Rb) #2217	1∶5,000;1∶10,000	5% Milk/TBST
pS6K1 T389	Cell Signal (Rb) #9234	1∶2,500;1∶4,000	Block in 5% milk, probe in 5% BSA(probe for pS6K1 prior to total S6K1)
N-terminal S6K1	Cell Signal (Rb) #2708	1∶1,1000;1∶2,000	5% Milk/TBST (used everywhereexcept fig. 4E panel 2)
C-Terminal S6K1	Cell Signal (Rb) #9202	1∶2,500;1∶4,000	5% BSA/TBST
TSC1	Cell Signal (Rb) #4906	1∶1,000;1∶1.000	5% Milk/TBST
TSC2	Cell Signal (Rb) #4308	1∶5,000;1∶10,000	5% Milk/TBST
Goat anti-Mouse IgG –HRPSecondary	Santa Cruz #sc2005		5% Milk/TBST
Anti-rabbit IgGSecondary	Cell Signal (Rb) #7074		5% Milk/TBST

### Plasmid and Transfection

Transfections were formed using PolyJet transfection reagent (SignaGen) according to the manufacturer’s protocol. Vectors and their sources are listed in Table 2.

**Table 2:** List of vectors.

Vectors	Source-Details
**CAG promoter**	
Constitutively active Rheb = Rheb^CA^	Dr. Hanada, Tokyo [49]: S16H mutant
Constitutively active AKT = AKT^CA^	HA-tagged myristoylated AKT
GST-tagged murine FMRP = GST-FMRP	Dr. Xinyu Zhao, University of Wisconsin with additional modifications as follows: S499A and S499D
**CMV promoter**	
Dominant negative TSC2ΔRL = TSC^DN^	Drs. LJ Field, Indiana University-Purdue Universityand K. Pasumarthi, Dalhousie University - Halifax, Nova Scotia [50]: Mutant bears deletions of AA 81-102 and AA 1679–1742
Hyperactive mTOR = mTOR^HYPER^	pCDNA3.1 vector, Addgene [51]: mutation E2419K making it insensitive to TSC regulation, yet still sensitive to rapamycin

### Pharmacological Agents

Rapamycin (Cat. No. tlrl-rap, InvivoGen), PF-470867 (Symansis), Bisindolylmaleimide V (B5) (Cat. No. ALX-270-053, Enzo Life Sciences), and okadaic acid (Cat. No. ICN15897310, MP Biomedicals) were used as indicated in the text. Okadaic acid was dissolved in ethanol (vehicle 2) for N2a cell culture experiments and DMSO when used to supplement lysis buffers. (S)-DHPG was purchased from Tocris and diluted in water.

### Kinase Assay with Recombinant (r) FMRP

Kinase assays were performed by Kinexus (Canada). Recombinant FMRP S500 and S500D were generated as previously described [23].

### Neuro2a (N2a) Cell Culture

In general, N2a cells were cultured in complete media (Dulbecco’s Modified Eagle Medium (DMEM) (Gibco 11965-092), 5% fetal bovine serum (FBS) (Gibco 16140-071), and 1% Penicillin-Streptomycin (Gibco 15140-122) in a 37°C incubator at 5% CO_2_. When cells reached approximately 70% confluence in six-well plates, the medium was replaced with pre-warmed complete media and treatment was begun one hour later. For *in vitro* experiments, individual treatments and transfections were performed between two and six times prior to performing the combined experiments represented in figures 2 and 3 (N = 4 for each condition in *in vitro* experiments). Cell lysis was performed on ice. Cells were rinsed twice with ice-cold 1X phosphate-buffered saline Laemmli and lysed in N2a lysis buffer (RIPA, 1x HALT protease/phosphatase inhibitor cocktail, 8U/10ml DNase I, 100nM okadaic acid). Cells were then scraped from the wells and lysates centrifuged at 16,000 RCF for 20 minutes at 4°C. The supernatant was added to 6X Laemmli sample buffer to a final concentration of 1X sample buffer and boiled for 5 minutes at 99°C.

### Immunoblotting

All western blots were performed using 10% tris-gylcine gels and protein transferred to PVDF membranes according to a standard wet transfer protocol. In cases where cross-blot normalization was required a standard sample was loaded on each gel to account for inter-gel variability. In general, the optimal linear range for each antibody was determined using the appropriate sample type (P2, whole cell etc.) prior to experimental immunoblot assays. In some cases, limited linear ranges were run on the same gel (that is 80% and 120% of a control sample were loaded in end lanes) to ensure detectability of minor changes in protein signals. Densitometry was performed using Image J without background correction or rolling ball adjustments. All phospho-protein signals were normalized to total protein signals from the same blot. For phospho-proteins, adequate removal of phospho-antibody was verified by probing with secondary alone after stripping the membrane. Raw ratios of phospho-protein normalized to total protein or total protein normalized to loading control (generally ERK unless otherwise stated) were calculated in Microsoft Excel.

### Statistical Analysis

Statistical analysis was performed on raw densitometric ratios using GraphPad 6. For data presentation, values were normalized to control data such that control groups were always = 1. For *in vitro* experiments, the data is represented as the % change from the control lane on the same membrane; as such, control lanes are without error bars. Statistical significance was determined using Mann-Whitney U, one-way ANOVA or two-way ANOVA using Dunnett’s and Turkey’s post-hoc tests where indicated. P<0.05 was considered significant. Data are shown as mean ± standard of the mean (SEM) unless otherwise specified.
